# Point-of-Care Lung Ultrasound for Differentiating COVID-19 From Influenza

**DOI:** 10.7759/cureus.21116

**Published:** 2022-01-11

**Authors:** Andrew J Goldsmith, Ahad Al Saud, Nicole M Duggan, Irene W Ma, Calvin K Huang, Onyinyechi Eke, Tina Kapur, Sigmund Kharasch, Andrew Liteplo, Hamid Shokoohi

**Affiliations:** 1 Emergency Medicine, Brigham and Women's Hospital, Harvard Medical School, Boston, USA; 2 Emergency Medicine, Massachusetts General Hospital, Harvard Medical School, Boston, USA; 3 Internal Medicine, University of Calgary, Calgary, CAN; 4 Emergency Medicine, Brigham and Women’s Hospital, Harvard Medical School, Boston, USA

**Keywords:** lung ultrasound, coronavirus, covid-19, influenza, point-of-care ultrasound, ultrasound

## Abstract

Background and objectives

Patients infected with influenza and COVID-19 exhibit similar clinical presentations; thus, a point-of-care test to differentiate between the diseases is needed. Here, we sought to identify features of point-of-care lung ultrasound (LUS) that can discriminate between influenza and COVID-19.

Methods

In this prospective, cross-sectional study, LUS clips of patients presenting to the emergency department (ED) with viral-like symptoms were collected via a 10-zone scanning protocol. Deidentified clips were interpreted by emergency ultrasound fellows blinded to patients’ clinical context and influenza or COVID-19 diagnosis. Modified Soldati scores were calculated for each lung zone. Logistic regression identified the association of pulmonary pathologies with each disease.

Results

Ultrasound fellows reviewed LUS clips from 165 patients, of which 30.9% (51/165) had confirmed influenza, 33.9% (56/165) had confirmed COVID-19, and 35.1% (58/165) had neither disease. Patients with COVID-19 were more likely to have irregular pleura and B-lines in all lung zones (p<0.01). The median-modified Soldati score for influenza patients was 0/20 (IQR 0-2), 9/20 (IQR 2.5-15.5) for COVID-19 patients, and 2/20 (IQR 0-8) for patients with neither disease (p<0.0001). In multivariate regression analysis adjusted for age, sex, and congestive heart failure (CHF), the presence of B-lines (OR = 1.29, 95% CI 1.09-1.53) was independently associated with COVID-19 diagnosis. The presence of pleural effusion was inversely associated with COVID-19 (OR = 0.09, 95% CI 0.01-0.65).

Conclusions

LUS may help providers preferentially identify the presence of influenza versus COVID-19 infection both visually and by calculating a modified Soldati score. Further studies assessing the utility of LUS in differentiating viral illnesses in patients with variable illness patterns and those with variable illness severity are warranted.

## Introduction

Each year, respiratory viruses, including influenza, run rampant throughout the global community. The Centers for Disease Control and Prevention (CDC) estimates that in 2018-2019 alone, more than 40 million people in the United States were infected with influenza, leading to more than 60,000 deaths [1]. In March 2020, Coronavirus disease 2019 (COVID-19) was deemed a global pandemic [2]. Given the distinct differences in transmissibility, clinical course, mortality, and potential treatment options, accurate and early differentiation between diseases such as influenza and COVID-19 is crucial for both patients and clinicians worldwide [3,4].

Patients with influenza and COVID-19 often present with similar symptoms, including cough, sore throat, myalgias, fatigue, fever, and shortness of breath [3]. Thus, differentiating these diseases based on clinical context alone can be challenging. Polymerase chain reaction (PCR) serves as the gold-standard confirmatory testing for both SARS-CoV-2 and influenza infections. However, testing may be inaccessible, time-consuming, and frequently insufficiently sensitive to be used as a rapid screening tool [5,6]. Although computed tomography (CT) imaging can accurately detect pulmonary disease, its role in the pandemic has been inconsistent due to challenges associated with scanner availability, unnecessary radiation exposure, and the potential for exposing additional health care personnel and patients to the virus via this communal imaging resource [7]. Further, transport to a CT scanner ideally requires a degree of clinical stability that some patients with COVID-19 lack.

Point-of-care ultrasound (POCUS) is a bedside diagnostic imaging modality that has become a critical tool for assessing acute dyspnea in the emergency department (ED) [8-10]. Well-established POCUS scanning protocols for evaluating dyspnea, such as the bedside lung ultrasound in emergency (BLUE)-protocol, are now primary steps in ED clinical workups [11]. As such, it has been widely demonstrated that emergency physicians can achieve high-quality cardiac and pulmonary images on POCUS to make accurate diagnoses. Lung ultrasound (LUS) demonstrates a higher sensitivity than chest radiography in detecting pulmonary pathologies such as pleural effusion, alveolar consolidation, pneumothorax, and interstitial syndrome [12-16]. Given its value in assessing lung pathology, LUS has evolved as an important tool to evaluate patients with suspected COVID-19 [17-20]. Among other roles, LUS has been broadly explored for its use in the diagnosis, triage, home monitoring, and pre-hospital evaluation of patients with COVID-19 [20-24].

While there has been ample discussion surrounding the use of LUS in both influenza and COVID-19, no study has evaluated whether LUS alone can differentiate between the two respiratory infections in patients presenting to the ED with undifferentiated dyspnea and viral-like illness [25-27]. The goal of this study was to determine whether a scoring system based on specific pathological LUS findings can help blinded physician reviewers differentiate between COVID-19 and influenza.

## Materials and methods

Study design and setting 

This was a cross-sectional study that took place in the ED of an urban academic teaching hospital. The hospital serves as a level I trauma center with an annual ED patient volume of approximately 120,000. The hospital is home to a four-year emergency medicine residency training program. This research was approved by the Massachusetts General Brigham Institutional Review Board (IRB) initially to enroll patients with influenza with the goal of defining LUS characteristic findings in this population (protocol number 2019P000189); however, when COVID-19 became more pervasive in EDs across the United States, IRB approval was amended under special circumstances to also enroll patients infected with COVID-19. All patients provided informed consent prior to study enrollment.

Selection of participants 

Patients 18 years of age or older who presented to the ED between December 2019 and September 2020 with chief complaints suggestive of a viral-like illness such as "cough," "fever," "sore throat," "shortness of breath," "myalgias," and "fatigue" were prospectively enrolled. These patients were identified via a combination of chief complaints assigned at triage as one of the terms above, as well as a referral from the clinical care team for patients who may be exhibiting "flu-like symptoms." While data for patients infected with influenza were collected throughout the study period (December 2019-September 2020), data collection for patients infected with severe acute respiratory syndrome coronavirus 2 (SARS-CoV-2) began in March 2020. The original study was designed to identify LUS findings characteristic of patients infected with influenza. Thus, the recruitment protocol was not changed between the original study design and the modified design. Additional inclusion criteria included patients who were being tested for influenza or SARS-CoV-2 via PCR in the ED. By March 2020, all patients were tested for both influenza and SARS-CoV-2. There were no exclusion criteria other than age. Patients were recruited via convenience sample when study investigators were present and available for enrollment. Based on PCR results, patients fell into one of three groups: patients diagnosed with (1) COVID-19, (2) influenza, or (3) other, which was made up of all patients who ultimately tested negative for both influenza and COVID-19 by PCR.

Data collection 

All POCUS scanning procedures were performed by trained providers (four emergency medicine attending physicians, four emergency medicine ultrasound fellows, two emergency medicine residents, and five emergency medicine physician assistants). All providers had experience with POCUS and additionally received one hour of standardized training in LUS image acquisition prior to participating in the study. LUS examinations were performed using a curvilinear 5-2 MHz transducer on the Mindray TE-7 (Mindray North America, Mahwah, NJ), Mindray M9 (Mindray North America), or GE Venue Go (General Electric, Boston, MA).

**Figure 1:**
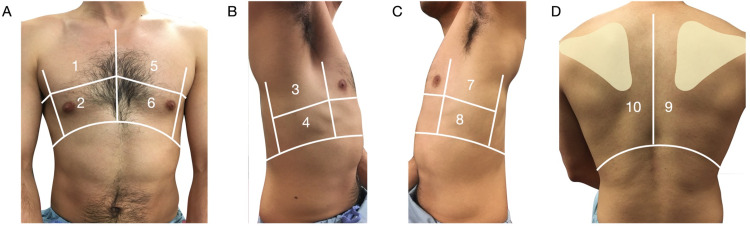
Ten-zone lung scanning protocol. (A) The anterior chest lung zones consistent of four zones with zones 1 and 2 representing anterior right chest and zones 5 and 6 representing the anterior left chest. (B) The right midaxillary line consisted of zone 3 (superior) and zone 4 (inferior). (C) The left midaxillary line consisted of zone 7 (i.e., superior) and zone 8 (i.e., inferior). (D) Zones 9 and 10 were posterior medial scapula lung zones of the right and left lungs, respectively.

All recruited patients underwent a complete LUS examination using an institutional standard ten-zone protocol (Figure 1). Six-second video clips were obtained for each of the ten zones. All LUS examinations (i.e., ten zones/patient) were de-identified and included in the final analysis. Retrospective chart review later identified patients who tested positive for either influenza or COVID-19 by PCR, or negative for both on their index ED visit. 

LUS interpretation**

Four emergency ultrasound fellows blinded to patients’ clinical presentation, date of presentation, and final diagnoses were randomly divided into two groups of two fellows in each group. Fellows groups retrospectively reviewed all ten deidentified video clips for each patient. Each fellow group (two fellows together) independently assessed and scored each zone for the presence or absence of common features of LUS, including A-lines, B-lines, irregular pleural lines, subpleural consolidations, and pleural effusion. Scores were independently recorded by each fellow group on a standardized electronic scoresheet. A clip was identified as positive for B-lines if three or more discrete or confluent B-lines were present in a six-second clip [11]. An irregular pleural line was defined as any indent in the continuous pleural line. Subpleural consolidations were defined as consolidations <0.5 cm with associated pleural line abnormalities and/or B-lines [11,19]. In the event of disagreement between the two fellow group interpretations for the presence of pathology on LUS (i.e., split interpretations by fellows on whether pathology such as B-lines, A-lines, irregular pleura, etc., was present), the gold standard interpretation was determined by an emergency medicine attending with fellowship training in emergency ultrasound.

**Figure 2:**
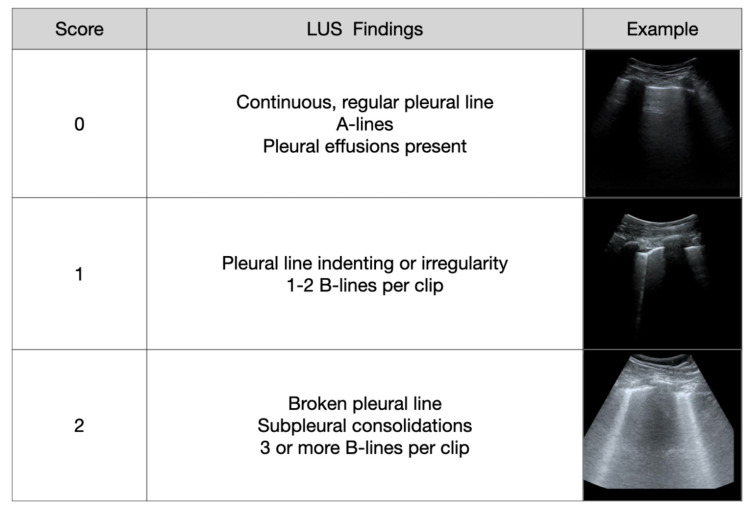
Modified Soldati scoring system used to assess lung ultrasound. LUS: lung ultrasound.

Based on each video clip's collated findings by all groups and the expert attending reviewer where needed, an LUS severity score was calculated using a standardized modified scoring system originally described by Soldati for COVID-19 (modified Soldati Score, Figure 2) [19]. One lung zone could receive a maximum of two points. Individual lung zone scores were then summed up to generate a total patient lung severity score. A patient could receive a maximum severity score of 20 points (e.g., 2 points per each of the 10 lung zones).

Data analysis and statistics 

The REDCap electronic data capture system was used to collect and manage study data. Frequency and percentages were used to summarize categorical variables. Continuous normal and non-normal data were summarized using the mean and standard deviation or median and interquartile range, respectively. Non-normal continuous variables amongst the three groups were compared using Kruskal Wallis tests, with post-hoc Dwass-Steel-Critchlow-Fligner analyses performed for multiple comparisons.

The lung zones and patient LUS scores were compared using Fisher exact tests. Logistic regression analyses were used to evaluate the association of LUS findings with COVID-19 diagnosis. Significant predictors (p<0.05) were retained in the multivariate model, along with adjustments for age, gender, and past medical history of congestive heart failure (CHF) in the multivariate regression model. All analyses were performed using the full dataset. A receiver operating characteristic (ROC) analysis was performed with a diagnostic accuracy of COVID-19 estimated with the area under the curve (AUC) statistic for all seven variables used in the logistic regression. A two-sided p-value of <0.05 was considered statistically significant. All analyses were performed using SAS version 9.4 (SAS Institute Inc., Cary, NC) and IBM SPSS software (version 26, IBM SPSS, Armonk, NY).

## Results

Characteristics of study subjects 

**Figure 3:**
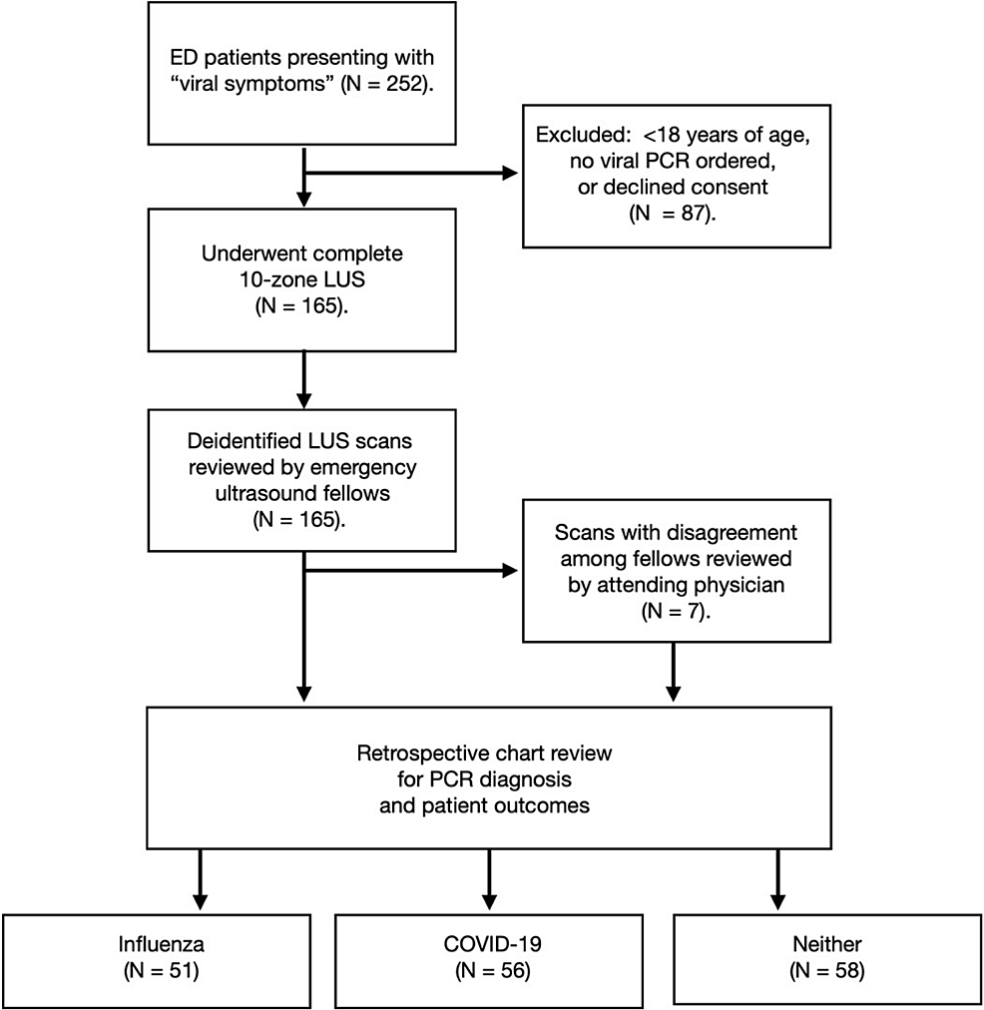
Flow diagram of patient enrollment. ED: emergency department; PCR: polymerase chain reaction; LUS: lung ultrasound.

In total, 165 patients were enrolled, of whom 30.9% (51/165) had influenza, 33.9% (56/165) had COVID-19, and 35.2% (58/165) had neither (Figure 3). No patients had a dual infection of both influenza and COVID-19. Of the 165 patient scans reviewed, the findings from seven patients required arbitration by an emergency medicine attending with emergency ultrasound training due to disagreement in interpretations between fellow groups.

The group of patients in the influenza group was significantly younger (an average of 50.6 years) compared to the COVID-19 group (an average of 59.6 years) and the negative COVID-19 and flu patients (61.4 ± 22.4, p=0.026). Further, the influenza group (10.4%) had statistically fewer patients with a past medical history of CHF patients than the COVID-19 group (30.4%) and the negative group (24.1%, p=0.046; Table 1).

**Table 1:** Patient demographics for influenza, COVID-19 PCR-positive, and PCR negative patients. PCR: polymerase chain reaction; BMI: body mass index; DVT: deep vein thrombosis; PE: pulmonary embolism; COPD: chronic obstructive pulmonary disease; ED: emergency department; BP: blood pressure; COVID-19: Coronavirus disease 2019.

Variable	All patients N=165	Influenza positive N=51	COVID-19 positive N=56	Negative COVID-19/Influenza N=58	P-value
Sex					
Male	73 (44.2%)	22 (43.1%)	27 (48.2%)	24 (41.4%)	0.750
Female	92 (55.8%)	29 (56.9%)	29 (51.8%)	34 (58.6%)	
Age (years)	57.1 ± 22.1	50.6 ± 22.9	59.6 ± 19.4	61.4 ± 22.4	0.026
BMI (kg/m^2^)	28.6 ± 16.6	29.2 ± 28.7	29.6 ± 7.4	27.0 ± 7.1	0.668
Comorbidities					
Hypertension	90 (54.5%)	21 (41.2%)	31 (55.4%)	38 (65.5%)	0.069
Diabetes mellitus	41 (24.8%)	7 (13.7%)	20 (35.7%)	14 (24.1%)	0.031
Congestive heart failure	36 (21.8%)	5 (9.8%)	17 (30.4%)	14 (24.1%)	0.032
Coronary artery disease	40 (24.2%)	7 (13.7%)	16 (28.6%)	17 (29.3%)	0.108
DVT/PE	15 (9.1%)	3 (5.9%)	7 (12.5%)	5 (8.6%)	0.487
COPD	27 (16.4%)	6 (11.8%)	6 (10.7%)	15 (25.9%)	0.052
Initial ED vital signs					
Systolic BP (mmHg)	129.4 ± 29.8	121.9 ± 22.5	132.1 ± 29.4	135.1 ± 29.8	0.017
Heart rate (beats per minute)	96.1 ± 25.3	104.7 ± 27.6	94.3 ± 22.7	92.1 ± 20.9	0.073
Respiratory rate (breaths per minute)	22.4 ± 6.9	21.6 ± 4.7	24.1 ± 8.2	21.8 ± 6.3	0.065
Temperature (ºC)	37.6 ± 9.0	37.2 ± 0.9	37.3 ± 9.2	38.9 ± 11.2	0.339
Room air O_2 _saturation (%)	91.2 ± 16.2	92.6 ± 14.4	91.9 ± 9.6	90.9 ± 18.4	0.926
ED disposition					
Hospital floor admission	116 (70.3%)	29 (56.9%)	45 (80.3%)	42 (72.4%)	0.027
ICU admission	25 (15.2%)	2 (3.9%)	15 (26.7%)	8 (13.8%)	0.004

Main results 

**Figure 4:**
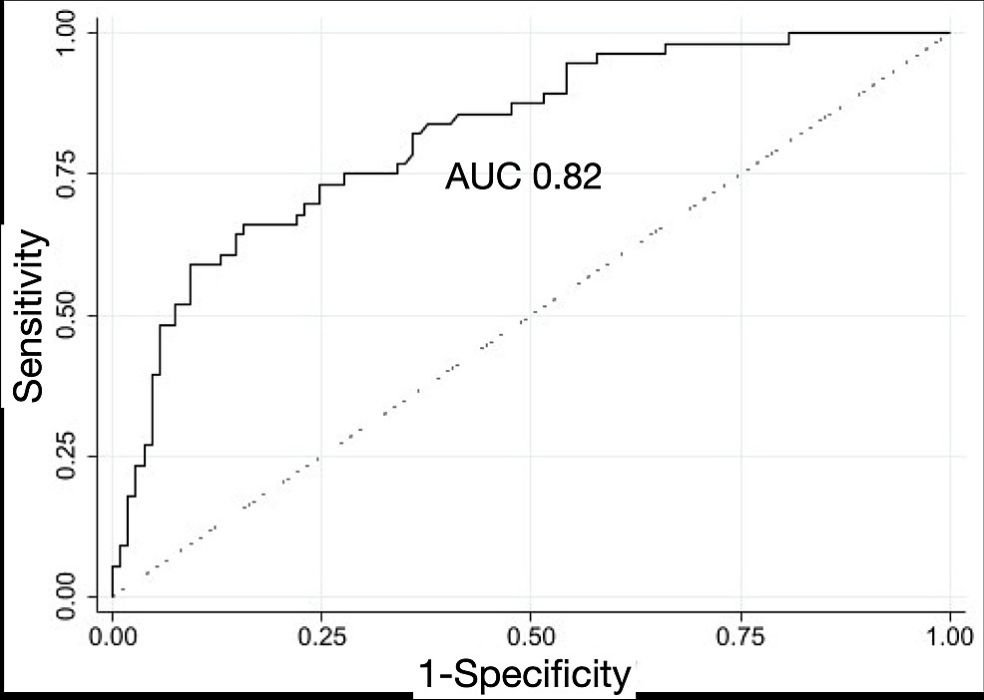
Receiver operator characteristic curve of multivariate model with COVID-19 as the diagnostic outcome. AUC: area under the curve.

When comparing LUS from patients with COVID-19 to patients with influenza, COVID-19 LUS had a higher incidence of irregular pleura, subpleural consolidations, and B-lines in all 10 lung zones. Pleural effusion was seen more often in influenza LUS as compared to COVID-19 LUS (p = 0.018). Univariate logistic regression analyses demonstrated a significant association of irregular pleura (OR = 1.36, 95% CI 1.17-1.59, p < 0.0001), sub-pleural consolidation (OR = 1.62, 95% CI 1.26-2.08, p = 0.0002), pleural effusion (OR = 0.14, 95% CI 0.02-0.83, p = 0.030), and B-lines (OR = 1.29, 95% CI 1.16-1.43, p < 0.0001) with COVID-19 (Table 2). When adjusted for age, gender, and prior history of CHF in the multivariate model, B-lines remained significant (OR = 1.29, 95% CI 1.09-1.53, p = 0.003) for COVID-19, as did the presence of pleural effusion (OR = 0.09, 95% CI 0.01-0.65, p = 0.018). This seven-variable model demonstrated an area under the curve value of 0.82 (Figure 4).

**Table 2:** Regression models using COVID-19 as the diagnostic outcome. *Each variable represents one lung zone. CHF: congestive heart failure.

Variable*	OR	95% CI	P-value
Univariate regression			
Irregular pleura*	1.36	1.17–1.59	<0.0001
Subpleural consolidation*	1.62	1.26–2.08	0.0002
B-lines*	1.29	1.16–1.43	<0.0001
Pleural effusion*	0.14	0.02–0.83	0.030
CHF	2.07	0.97–4.39	0.060
Age	1.01	0.99–1.02	0.29
Sex (Ref: female)	1.28	0.67–2.44	0.46
Adjusted for CHF, age, and gender			
Multivariate regression			
Irregular pleura*	1.02	0.78–1.34	0.88
Subpleural consolidation*	1.24	0.81–​​​​​​​1.88	0.32
B-lines*	1.29	1.09–​​​​​​​1.53	0.003
Pleural effusion*	0.09	0.01–​​​​​​​0.65	0.018
CHF	1.52	0.57–​​​​​​​4.08	0.40
Age	1.00	0.98–​​​​​​​1.02	0.96
Gender (male, ref: female)	1.41	0.65–​​​​​​​3.07	0.38

LUS from COVID-19 patients received a significantly higher median Modified Soldati Score of 9 (IQR 2.5-15.5) when compared to LUS from influenza patients, which received a score of 0 (IQR 0-2; p < 0.0001), and to LUS from patients in the negative group, which received a median score of 2 (IQR 0-8, p = 0.0012; Table 3).

**Table 3:** Modified Soldati score by diagnosis. IQR: interquartile range; COVID-19: Coronavirus disease 2019.

Diagnosis	Modified Soldati Score (IQR)	P-value
Influenza	0 (0–2)	<0.0001
COVID-19	9 (2–15.5)	
Neither	2 (0–8)	

**Figure 5:**
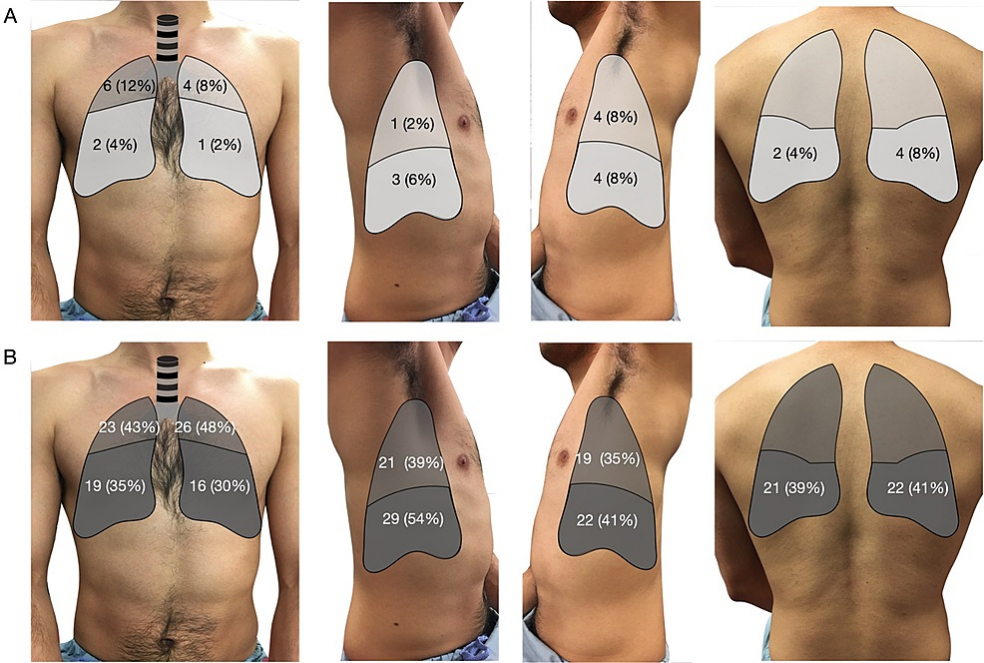
B-lines heat map for patient with influenza (A), and COVID-19 (B). Differences in the presence of B-lines between the two infections were statistically significant across all lung zones, respectively.

When comparing COVID-19 to influenza, COVID-19 LUS had a higher incidence of irregular pleura, subpleural consolidations, and B-lines in all 10 lung zones. Pleural effusion was seen more often in influenza as compared to COVID-19 LUS (p = 0.018, Table 2). Univariate logistic regression analyses demonstrated the significant association of irregular pleura, sub-pleural consolidation, and B-lines with COVID-19. In the multivariate model, only B-lines remained significant (OR 1.29, 95% CI 1.09-1.53, p = 0.003) for COVID-19, after adjusting for the effects of the other LUS findings. This model demonstrated an area under the curve of 0.82. Further, B-lines had a higher incidence in all 10 lung zones in LUS from COVID-19 as compared to influenza (Figure 5).

## Discussion

In this study, we identified that patients with COVID-19 in the first wave of the pandemic demonstrated a higher lung severity score compared to both patients with influenza and PCR-negative patients presenting with undifferentiated "flu-like symptoms." In a recent multicenter observational study, LUS combined with clinical presentation and assigned disease phenotype was used to try to predict the likelihood of a COVID-19 diagnosis as confirmed by PCR [28]. The authors identified that in cohorts that demonstrated more severe LUS findings, the sensitivity of LUS in identifying COVID-19 infection was >90% and increased when combined with increasing severity of clinical symptoms. Taken together with these findings, our work suggests that when clinically assessing a patient with "flu-like symptoms," LUS may help clinicians efficiently and effectively differentiate between influenza and COVID-19 through either visual assessment of LUS clip severity or through applying a formalized scoring system. Combining either subjective or objective clinical illness data into our prediction may strengthen the value of LUS in differentiating COVID-19 from influenza; however, this needs to be investigated further in subsequent works.

The B-lines were the predominant feature seen in COVID-19 LUS compared to LUS from influenza or the "other" patient cohorts. As the presence of B-lines is one of several components of the LUS-modified Soldati Score, it logically follows that COVID-19 patients demonstrated higher severity scores when compared to influenza patients. This scoring system may help clinicians differentiate between influenza and COVID-19. We postulate that a combination of LUS and clinical parameters such as vital signs can help clinicians differentiate between COVID-19 and other conditions which can also present similarly with acute dyspnea, such as bacterial pneumonia, acute heart failure exacerbation, or pulmonary embolism; however, this has yet to be elucidated. The more zones that have B-lines, subpleural consolidations, and irregular pleura resulted in a higher score. As the latter findings are rarer in alternative causes of dyspnea, it is likely that a simple scoring system such as the one developed here could be of high utility for healthcare workers on the front line. This could hold especially true in low-resource settings where PCR testing availability remains limited. Larger prospective studies would be needed to explore this further. 

Here, we identified that B-lines are associated with the diagnosis of COVID-19 independent of other typical findings such as irregular pleural and subpleural consolidations. Importantly, the increasing number of lung zones with B-lines was associated with an increased likelihood of COVID-19 diagnosis. Though likely useful in differentiating COVID-19 from influenza, it may be difficult to visually delineate diagnoses such as decompensated heart failure from COVID-19 based on B-lines alone. Interestingly, recently, a deep learning algorithm was trained to differentiate between the visually similar-appearing lung pathologies of COVID-19, non-COVID-19 respiratory distress syndrome, and non-infectious hydrostatic pulmonary edema based on the appearance of the B-lines on LUS alone [29]. This may suggest that adding an automated deep learning component to LUS when trying to differentiate similar-appearing pathologies such as COVID-19 and influenza may make for even more robust and accurate test characteristics and outcomes. 

In conjunction with B-lines, the distribution of LUS findings may be used to guide diagnostic evaluation. In COVID-19, LUS findings are predominantly observed in lateral and posterior distributions, a trend that is also seen in CT and chest X-rays [17-19]. While our data do not demonstrate statistical significance in the distribution of LUS findings in COVID-19 when compared with influenza, the posterior and lateral lung zones did have more findings overall when compared to the anterior zones in COVID-19 patients. These data suggest that the location of LUS findings may help clinicians differentiate COVID-19 from alternative diagnoses in patients presenting with "flu-like illness"; however, additional studies are needed to clarify this further.

Limitations

Importantly, one major limitation of this study relates to the clinical differences between our COVID-19 and influenza cohorts. Namely, our influenza cohort was significantly younger, and our COVID-19 cohort had a higher frequency of both hospital and ICU admissions. This suggests that our patients with COVID-19 were overall sicker or presented in more advanced disease states than our patients with influenza, and thus potentially demonstrated more exaggerated findings on LUS than what would have been seen in a population of less-ill patients. While we agree that during the enrollment period, the pathophysiology of COVID-19 was likely more severe than that of influenza, this is an important disease trend and does not necessarily discount the value of LUS in assessing undifferentiated patients presenting to the ED with flu-like illnesses. The next step to further clarify these trends could be to perform cohort analyses with matched patients with COVID-19 and influenza for illness severity. This severity of illness was representative of the patient population during the first wave of COVID-19 in the United States when this study was being performed. These findings may not necessarily be reproducible in subsequent populations that exhibit variable illness severity. As more COVID variants with more variable illness profiles become more pervasive, it will be critical to perform subsequent studies such as this to evaluate the true value or LUS in COVID-19 patients with lower illness severity.

Our COVID-19 cohort had a different profile of comorbidities, namely in this cohort there were more patients with a prior history of CHF. As LUS findings in older patients with CHF may be related to additional overlapping pathologies as opposed to viral infection alone, we did adjust our analysis to ensure our findings were not related to differences in the past medical history of CHF, age, or gender. We did not specifically exclude patients with additional acute pathologies such as bacterial pneumonia, acute pulmonary embolism, or acute CHF exacerbation from enrollment.

The data in this study were collected and analyzed at a single institution by providers with advanced ultrasound training and experience, thus potentially limiting the generalizability of our findings. However, in a retrospective review assessing agreement between learner and expert POCUS users, both image acquisition quality and image interpretation accuracy differed by POCUS application [30]. Here, LUS performed to assess for the presence of pneumothorax was the easiest application for obtaining satisfactory image quality, with only three LUS novice users producing an image quality of 95% of that of expert images. While authors did not assess learners' ability to acquire and identify images with LUS findings assessed here, such as B-lines or subpleural consolidations, this work does suggest that overall LUS is a relatively easy POCUS application for even novice learners to perform. Therefore, we predict our findings will appeal to a broad population of healthcare providers faced with assessing and accurately diagnosing patients with "viral illnesses." 

While both COVID-19 and influenza diagnoses were confirmed by PCR testing in all cases, it is possible that misclassification may have occurred in some cases. Repeat testing to confirm diagnoses may be helpful in some cases. In our cohort, we did not identify any confirmed cases of dual infection. It would be interesting to explore LUS findings in patients infected with both COVID-19 and influenza to further characterize the diagnostic utility of LUS. Similarly, it is uncertain how our results would perform in patients with underlying lung pathology such as interstitial lung disease. Further assessment in this patient cohort is warranted.

## Conclusions

LUS may play an important role in rapid bedside identification of viral infections and further in differentiating COVID-19 from influenza. Our data identified that in relatively ill cohorts, differences in LUS findings between patients with influenza and COVID-19 may include a higher presence of B-lines, fewer pleural effusions, and a higher modified Soldati Score in patients infected with COVID-19. Further studies are needed to determine if clinical history and exam, or the addition of deep learning automated interpretation, can contribute further to the diagnostic accuracy of LUS for COVID-19 and influenza across broader patient populations and clinical contexts.
